# Circulating sex hormone levels in relation to male sperm quality

**DOI:** 10.1186/s12894-020-00674-7

**Published:** 2020-07-17

**Authors:** Wei Zhao, Jun Jing, Yong Shao, Rong Zeng, Cencen Wang, Bing Yao, Dong Hang

**Affiliations:** 1 Reproductive Medical Center, Jinling Hospital Affiliated Medical School of Nanjing University, Nanjing, China; 2 Reproductive Medical Centre, Nanjing Jinling Hospital, Nanjing University School of Medicine, 305 Zhongshan East Road, Nanjing, 210002 Jiangsu China; 3 Department of Epidemiology and Biostatistics, School of Public Health, Nanjing Medical University, Nanjing, 211166 China

**Keywords:** Sex hormone, Luteinizing hormone, Sperm quality, Sperm motility, Sperm morphology

## Abstract

**Background:**

Although sex hormones play critical roles in spermatogenesis and sperm maturation, it remains inconclusive whether circulating sex hormones can serve as non-invasive biomarkers to improve the assessment of sperm quality.

**Methods:**

We systematically evaluated the association of various sex hormones in serum with sperm quality among 338 men in subfertile couples. Concentrations of luteinizing hormone (LH), follicle-stimulating hormone (FSH), total testosterone (TT), total estradiol (E2), and sex hormone-binding globulin (SHBG) were detected by chemiluminescent immunoassay. Free testosterone and estradiol were calculated using a validated algorithm. A generalized liner regression model controlling for lifestyle factors was used to evaluate the associations with sperm count, concentration, motility, and morphology.

**Results:**

After adjusting for age, body mass index, current smoking and alcohol drinking, LH, FSH, and TT levels were all inversely associated with sperm motility (all *P* for trend < 0.05); however, in mutual adjustment analysis, only LH remained an inverse association with sperm motility after adjusting for FSH and TT levels (*P* for trend = 0.04). Higher concentrations of LH were also associated with lower sperm progressive motility (*P* for trend = 0.04). Moreover, LH and FSH levels were both inversely associated with normal sperm morphology (*P* for trend = 0.04 and 0.02, respectively).

**Conclusions:**

Increased levels of LH are associated with poor sperm motility and morphology, suggesting that LH may play a central role in sperm maturation. Future studies are warranted to assess potential clinical utility of LH for risk stratification and tailed prevention of male infertility.

## Background

Over the past decades, infertility has become a serious health problem worldwide. Nearly 10–15% of couples at optimal reproductive age suffer from infertility [1], and 50% of cases are attributable to impaired sperm quality [2, 3]. In 2010, the WHO proposed a new manual for the laboratory examination of human semen, in which new reference values for semen characteristics were markedly lower than those reported in previous versions [4]. In addition to semen analysis, a blood test is commonly conducted to examine sex hormone concentrations for the males seeking fertility evaluation or treatment. Nevertheless, due to limited and inconsistent results from population-based studies, it is not well understood how variability in the levels of circulating sex hormones impact semen quality.

Sex hormones, such as luteinizing hormone (LH), follicle-stimulating hormone (FSH), estradiol (E2), testosterone, and sex hormone-binding hormone (SHBG), are demonstrated to play vital roles in spermatogenesis and sperm maturation [5, 6]. Several population-based studies, but not others [7–9], reported that circulating levels of sex hormones were associated with sperm concentration, motility, or morphology [10–12]. The exact reasons for this inconsistency remain unknown, but most of the studies were limited by small sample sizes and inadequate control for potential confounding (e.g., age, body mass index [BMI], smoking, and alcohol consumption). Moreover, because various sex hormones tend to be correlated with each other, statistical analysis is required to separate their independent effects. Therefore, current data are far from conclusive to guide clinical evaluation of semen quality. Further investigations are warranted to address the relationship between circulating sex hormones and sperm quality, which may provide a non-invasive tool to improve early detection and treatment of male infertility.

In this study, we systematically evaluated the association of various sex hormones in serum with sperm quality among 338 men in subfertile couples. We not only adjusted for the factors including age, BMI, smoking, and alcohol consumption, but also performed mutual adjustment for sex hormones. The results could reveal the independent association of aforementioned hormones, and provide the clues about the specific effect of each hormone on semen quality.

## Method

### Study population

This study included a cohort of men in subfertile couples who sought evaluation and treatment at Nanjing Jinling Hospital between August 2012 and June 2015. They were aged 18 to 50 years, and their partners had not conceived within 12 months after stopping use of contraception. All participants were asked to complete a questionnaire about occupation, lifestyle factors (e.g., alcohol consumption and smoking history), and medical and reproductive history. After undergoing physical examination, they provided semen specimens and fasting venous blood. Exclusion criteria included azoospermia or current use of exogenous hormones. Among 338 eligible participants, 305 (90.2%) had normal semen concentrations, 189 (55.9%) had normal sperm total motility, and 229 (67.8%) had normal sperm morphology, according to the 2010 WHO reference values. A total of 165 (48.8%) men had one or more parameter below the reference value. This study was approved by the Human Subject Committees of Nanjing Jinling Hospital, and written informed consent was obtained from each participant.

### Analysis of semen parameters

Semen specimens were collected through masturbation after 2–7 days of sexual abstinence according to the 2010 WHO guidelines. After liquefaction at 37 °C, total sperm count, sperm concentration, total motility, and progressive motility were analyzed using a computer-aided sperm analysis system (CFT-9201; Jiangsu Rich Life Science Instrument Co., Ltd., Nanjing, China). Sperm morphology was evaluated using Diff-Quik staining according to the manufacture’s protocol (Nanjing Xindi Biological Pharmaceutical Engineering Co., Ltd. Nanjing, China). At least 200 spermatozoa were counted for each specimen, and the experiments were performed at least twice.

### Determinations of serum sex hormones

Serum was isolated from blood samples by centrifugation at 3000 g for 5 min. Concentrations of LH, FSH, TT, E2, and SHBG were determined by chemiluminescent immunoassay with commercially available kits (Beckman Coulter, Inc., USA) and an automated Unicel Dxl 800 Access Immunoassay System (Beckman Coulter, Inc., USA). The lower detection limits were 0.2 IU/L for LH, 0.2 IU/L for FSH, 0.35 nmol/L for TT, 18 pmol/L for E2, and 0.33 nmol/L for SHBG. The intra-assay coefficients of variation (CV) for LH, FSH, TT, E2 and SHBG were all less than 5%, and the inter-assay CVs were all less than 8%.Free testosterone and estradiol were calculated using a validated method based on total testosterone or estradiol, SHBG, an assumed constant representing the normal albumin concentration, and the association constants for the binding of testosterone and estradiol to SHBG and albumin [13]. Calculated values for free testosterone and estradiol was shown to be highly correlated (*r* ≥ 0.85) with direct measurements of free hormone levels [14], and this method was commonly used in previous studies [15, 16].

### Statistical analysis

We calculated the partial Spearman correlation coefficients between each two of the hormones after adjusting for age at blood collection. Adjusted least-squares means (LSM) of semen parameters by tertiles of hormone concentrations were calculated using a generalized liner regression model controlling for age, BMI, current smoking and alcohol drinking status. In mutual adjustment analysis, all hormones associated with sperm quality were included in the multivariate models. We tested for linear trend across hormone categories by treating them as ordinal prediction in multivariate linear regression models. All data analyses were conducted using SAS version 9.4 (SAS Institute, Cary, NC). *P*-value < 0.05 was considered statistically significant.

## Result

### Study population

Table 1 shows the characteristics of 338 male participants and the concentrations of serum sex hormones. The mean age of participants was 28.8 years, and most of them had been married (81%). The proportions of current smoking and alcohol drinking were 48 and 63%, respectively.

**Table 1:** Basic characteristics of study participants^a^

Variable	*N* = 338
Age at blood draw (year)	28.8 ± 5.1
BMI (kg/m^2^)	24.0 ± 3.1
Married, %	81.1
Current smoking, %	47.9
Current alcohol drinking, %	63.3
Serum sex hormone	
Luteinizing hormone (IU/L)	4.6(2.8)
Follicle-stimulating hormone (IU/L)	4.5(2.3)
Total testosterone (nmol/L)	14.0(6.1)
Free testosterone (nmol/L)	0.3(0.1)
Total estradiol (pmol/L)	98.5(57.0)
Free estradiol (pmol/L)	1.7(1.1)
Sex hormone-binding hormone (nmol/L)	27.9(17.8)

^a^Mean ± SD is presented for continuous variables, percentage for categorical variables, and median value (interquartile range) for sex hormones

### Correlation between serum sex hormone

Before analyzing the association between sex hormones and sperm parameters, we evaluated the correlation between sex hormones (Table 2). According to age-adjusted Spearman correlation coefficients, moderate positive correlations (*r* = 0.22–0.55, all *P* < 0.001) were observed for LH and FSH, LH and total testosterone, total/free testosterone and total estradiol, total testosterone and SHBG. We also found moderate inverse associations of SHBG with free testosterone (*r* = − 0.32, *P* < 0.001) and free estradiol (*r* = − 0.24, *P* < 0.001), supporting the known role of SHBG in lowering free testosterone and estradiol.

**Table 2:** Age-adjusted Spearman correlation coefficient between serum sex hormones

Sex hormone	Luteinizing hormone	Follicle-stimulating hormone	Total testosterone	Free testosterone	Total estradiol	Free estradiol	Sex hormone-binding hormone
Luteinizing hormone	**–**						
Follicle-stimulating hormone	0.28^c^	**–**					
Total testosterone	0.24^c^	0.18^b^	**–**				
Free testosterone	0.16^b^	0.08	0.55^c^	**–**			
Total estradiol	0.02	−0.08	0.28^c^	0.22^c^	**–**		
Free estradiol	−0.03	− 0.13^a^	0.09	0.32^c^	0.94^c^	**–**	
Sex hormone-binding hormone	0.12^a^	0.11^a^	0.55^c^	−0.32^c^	0.08	−0.24^c^	**–**

^a^*P* < 0.05; ^b^*P* < 0.01; ^c^*P* < 0.001

### LH and FSH are associated with sperm motility and morphology

Table 3 shows the association between sex hormone levels and sperm total motility.. The adjusted LSM (95% CI) of sperm motility was calculated according to the tertiles of each sex hormone. We found that LH, FSH, and TT levels were all inversely associated with sperm motility (all *P* for trend < 0.05). In mutual adjustment analysis, only LH remained a statistically significant association with sperm total motility after further adjusting for FSH and TT (*P* for trend = 0.01). Compared to the lowest tertile of LH (adjusted LSM = 47.1%), the highest tertile group had decreased sperm total motility (adjusted LSM = 40.5%, *P* = 0.04).

**Table 3:** Association between serum sex hormone levels and sperm total motility^a^

Sex hormone	n	Sperm total motility (%)		
		Least-squares mean (95% CI)	*P*	*P* for trend
Luteinizing hormone				
Tertile 1	112	48.4(44.4, 52.3)	Ref	0.002
Tertile 2	114	43.9(40.1, 47.8)	0.12	
Tertile 3	112	39.4(35.4, 43.3)	0.002	
Luteinizing hormone^b^				
Tertile 1	101	47.1(42.9, 51.3)	Ref	0.04
Tertile 2	98	44.3(40.2, 48.5)	0.36	
Tertile 3	91	40.5(36.0, 44.9)	0.04	
Follicle-stimulating hormone				
Tertile 1	112	46.6(42.6, 50.5)	Ref	0.01
Tertile 2	114	45.5(41.6, 49.4)	0.71	
Tertile 3	112	39.6(35.6, 43.5)	0.01	
Follicle-stimulating hormone^b^				
Tertile 1	93	44.3(39.9, 48.6)	Ref	0.54
Tertile 2	97	45.7(41.5, 49.9)	0.65	
Tertile 3	100	42.4(38.1, 46.6)	0.54	
Total testosterone				
Tertile 1	96	48.2(43.8, 52.6)	Ref	0.03
Tertile 2	97	43.0(38.8, 47.2)	0.09	
Tertile 3	97	41.1(367, 45.5)	0.03	
Total testosterone^b^				
Tertile 1	96	47.3(42.9, 51.7)	Ref	0.14
Tertile 2	97	42.7(38.5, 46.9)	0.14	
Tertile 3	97	42.3(37.9, 46.8)	0.14	
Free testosterone				
Tertile 1	96	46.7(42.3, 51.0)	Ref	0.06
Tertile 2	97	44.9(40.7, 49.1)	0.56	
Tertile 3	97	40.7(36.5, 45.0)	0.06	
Total estradiol				
Tertile 1	113	44.2(40.3, 48.2)	Ref	0.60
Tertile 2	115	44.7(40.8, 48.6)	0.87	
Tertile 3	110	42.7(38.7, 46.7)	0.59	
Free estradiol				
Tertile 1	96	43.5(39.3, 47.7)	Ref	0.339
Tertile 2	97	48.3(44.0, 52.5)	0.12	
Tertile 3	97	40.5(36.3, 44.7)	0.32	
Sex hormone-binding hormone				
Tertile 1	112	45.4(41.2, 49.6)	Ref	0.95
Tertile 2	113	41.3(37.4, 45.3)	0.18	
Tertile 3	113	44.9(40.9, 49.0)	0.88	

^a^General linear models were adjusted for age at sample collection, BMI (continuous), current smoking (yes or no), and current alcohol consumption (yes or no). *P* for trend was calculated by treating hormone categories as ordinal predictors in multivariate linear regression models

^b^Mutual adjustment for hormones associated with sperm motility in multivariate linear regression models

We further analyzed the association between sex hormone levels and sperm progressive motility, i.e. swimming in a straight line or in a large circle, which is necessary for fertilization. In Table 4, serum LH levels were inversely associated with sperm progressive motility. Compared to the lowest tertile of LH (adjusted LSM = 30.0%), the highest tertile group showed decreased progressive motility (adjusted LSM = 26.5%, *P* = 0.04).

**Table 4:** Association between serum sex hormone levels and sperm progressive motility^a^

Sex hormone	n	Progressive motility (%)		
		Least-squares mean (95% CI)	*P*	*P* for trend
Luteinizing hormone				
Tertile 1	112	30.0(27.6, 32.4)	Ref	0.04
Tertile 2	114	27.8(25.5, 30.2)	0.20	
Tertile 3	112	26.5(24.1, 28.9)	0.04	
Follicle-stimulating hormone				
Tertile 1	112	28.9(26.5, 31.3)	Ref	0.14
Tertile 2	114	29.1(26.7, 31.4)	0.94	
Tertile 3	112	26.4(24.0, 28.8)	0.14	
Total testosterone				
Tertile 1	96	29.6(26.9, 32.3)	Ref	0.22
Tertile 2	97	28.3(25.7, 30.8)	0.48	
Tertile 3	97	27.1(24.4, 29.9)	0.23	
Free testosterone				
Tertile 1	96	30.3(27.6, 32.9)	Ref	0.05
Tertile 2	97	28.2(25.7, 30.8)	0.28	
Tertile 3	97	26.5(23.9, 29.2)	0.06	
Total estradiol				
Tertile 1	113	28.4(26.0, 30.7)	Ref	0.69
Tertile 2	115	28.3(26.0, 30.7)	0.98	
Tertile 3	110	27.7(25.2, 30.1)	0.69	
Free estradiol				
Tertile 1	96	28.16(25.6, 30.7)	Ref	0.35
Tertile 2	97	30.5(27.9, 33.1)	0.21	
Tertile 3	97	26.4(23.8, 28.9)	0.34	
Sex hormone-binding hormone				
Tertile 1	112	27.6(25.0, 30.1)	Ref	0.26
Tertile 2	113	27.2(24.8, 29.6)	0.85	
Tertile 3	113	29.6(27.1, 32.0)	0.29	

^a^General linear models were adjusted for age at sample collection, BMI (continuous), current smoking (yes or no), and current alcohol consumption (yes or no). *P* for trend was calculated by treating hormone categories as ordinal predictors in multivariate linear regression models

In Table 5, we observed that LH and FSH were both inversely associated with the proportion of normal sperm morphology. Compared with the lowest tertile of LH (adjusted LSM = 6.8%), the highest tertile group had a lower proportion of normal sperm morphology (adjusted LSM = 6.0%, *P* = 0.01). Moreover, the highest tertile of FSH showed a lower proportion of normal sperm morphology (adjusted LSM = 6.0%, *P* = 0.01) than the lowest tertile (adjusted LSM = 7.0%). The associations for LH and FSH remained statistically significant in mutual adjustment analysis.

**Table 5:** Association between serum sex hormone levels and the proportion of normal sperm morphology^a^

Sperm parameters	n	Normal sperm morphology (%)		
		Least-squares mean (95% CI)	*P*	*P* for trend
Luteinizing hormone				
Tertile 1	112	6.8(6.5, 7.5)	Ref	0.01
Tertile 2	114	6.9(6.4, 7.3)	0.74	
Tertile 3	112	6.0(5.5, 6.5)	0.01	
Luteinizing hormone^b^				
Tertile 1	111	6.9(6.4, 7.4)	Ref	0.04
Tertile 2	111	6.8(6.4, 7.3)	0.87	
Tertile 3	109	6.1(5.6, 6.6)	0.04	
Follicle-stimulating hormone				
Tertile 1	112	7.0(6.5, 7.5)	Ref	0.01
Tertile 2	114	6.8(6.3, 7.3)	0.46	
Tertile 3	112	6.0(5.5, 6.5)	0.01	
Follicle-stimulating hormone^b^				
Tertile 1	109	7.0(6.5, 7.5)	Ref	0.02
Tertile 2	114	6.8(6.3, 7.2)	0.54	
Tertile 3	108	6.1(5.6, 6.6)	0.02	
Total testosterone				
Tertile 1	96	7.1(6.5, 7.6)	Ref	0.06
Tertile 2	97	6.8(6.2, 7.3)	0.41	
Tertile 3	97	6.3(5.7, 6.9)	0.06	
Free testosterone				
Tertile 1	96	6.7(6.1, 7.2)	Ref	0.94
Tertile 2	97	6.7(6.2, 7.2)	0.99	
Tertile 3	97	6.7(6.2, 7.3)	0.94	
Total estradiol				
Tertile 1	113	6.5(6.0, 7.0)	Ref	0.91
Tertile 2	115	6.8(6.3, 7.3)	0.50	
Tertile 3	110	6.6(6.1, 7.1)	0.92	
Free estradiol				
Tertile 1	96	6.5(5.9, 7.0)	Ref	0.49
Tertile 2	97	6.9(6.4, 7.4)	0.27	
Tertile 3	97	6.7(6.2, 7.3)	0.50	
Sex hormone-binding hormone				
Tertile 1	112	7.0(6.5, 7.6)	Ref	0.06
Tertile 2	113	6.5(6.0, 7.0)	0.14	
Tertile 3	113	6.3(5.8, 6.8)	0.06	

^a^General linear models were adjusted for age at sample collection, BMI (continuous), current smoking (yes or no), and current alcohol consumption (yes or no). *P* for trend was calculated by treating hormone categories as ordinal predictors in multivariate linear regression models

^b^Mutual adjustment for hormones associated with the proportion of normal sperm morphology in multivariate linear regression models

We also analyzed the association between sex hormone levels and total sperm count and sperm concentrations. We found that none of sex hormones was associated with total sperm count (All *P* for trend > 0.05) (Supplementary Table 1). Higher levels of LH and FSH appeared to be associated with lower sperm concentrations, but the trend tests were statistically non-significant (Supplementary Table 2).

In a sensitivity analysis by excluding participants with semen concentrations at the top and bottom 5%, the above results were essentially unchanged (data not shown).

## Discussion

This study showed that serum concentrations of LH were inversely associated with sperm motility and normal morphology, after adjusting for various lifestyle factors and other sex hormone levels. In addition, higher levels of FSH showed an independent association with a lower proportion of normal sperm morphology. No statistically significant association was observed between sex hormones and total sperm count or sperm concentrations. Our findings suggested that LH might play a central role in the sperm motility and morphology, supporting the utility of circulating LH levels as a biomarker for assessing sperm quality.

A few studies have investigated the relationship between circulating sex hormones and sperm parameters. *Kumanov* et al. found that serum concentrations of LH and FSH were inversely correlated with sperm count, motility, and morphology, while testosterone was not correlated [10]. Similarly, another study by *Meeker* et al. reported significant negative correlations of LH and FSH with sperm concentration, motility, and morphology; however, testosterone levels were significantly positively correlated with motility [11]. In contrast, two small studies reported that only FSH levels had a negative correlation with semen parameters, while LH and testosterone levels did not [7, 8]. Compared with those studies, the current study had relatively large sample size, included various sex hormones, adjusted for lifestyle factors, and performed mutual adjustment analysis. We found that LH, FSH, and TT were all inversely associated with sperm total motility; however, only LH had an independent association after adjusting for FSH and TT, supporting the central role of LH in sperm motility. Consistently, only LH levels were found to be inversely associated with sperm progressive motility. We also observed that LH and FSH were inversely associated with sperm morphology after mutual adjustment, suggesting that both of them are important for sperm to maintain normal morphology. To our knowledge, the current study represents a first attempt to disentangle independent effects of various sex hormones on sperm quality.

The primary role of LH in the male is to stimulate the production of testosterone by the Leydig cells which then, together with FSH, control spermatogonial cell formation and spermatogenesis in the Sertoli cells [17]. Early studies indicated that gonadal failure, a cause of infertility, was characterized by increased levels of LH and FSH [18]. Male patients with idiopathic oligozoospermia were also found to have a higher mean LH pulse frequency than the controls [19]. The increase in LH or FSH concentrations may reflect that the testicles have insufficient capacity for normal spermatogenesis. In the current study, we observed a suggestive association between LH/FSH and sperm concentration. On the other hand, LH may affect fructose utilization, glucose oxidation, and adenyl cyclase activity in sperm, which are important means by which spermatozoa derive energy for motility [20]. The acquisition of sperm motility occurs during sperm maturation in the epididymis, and LH receptors have been detected in epididymal epithelium [21, 22]. Moreover, in the absence of LH, addition of TT and FSH can only partly rescue the phenotype of abnormal sperm [23]. These evidences are in support of our findings, suggesting a critical role of LH in sperm motility. Although recent studies support that LH may be also implicated in sperm morphology [24, 25], specific mechanisms remain unknown. More functional research is warranted to clarify the current results.

This study has several strengths, including relatively large sample size, inclusion of various sex hormones, adjustment for potential confounding, and mutual adjustment analysis to determine independent effects. However, our analysis was cross-sectional, which limited the ability to make causal inference. Additionally, only a single measurement of circulating hormones was available that may not represent long-term levels. Prospective studies with repeated assessment of circulating hormones are necessary to validate our findings. Finally, semen parameters are variable within individuals over time and one semen sample may not well reflect a man’s long-term values. However, prior studies have reported no significant differences between the first semen sample and the remaining replicates, suggesting one semen sample may suffice to identify average differences in semen quality between individuals [26, 27].

## Conclusions

In summary, our study demonstrates that circulating LH is inversely associated with sperm motility and morphology, suggesting that LH might act as a major regulator in sperm maturation. Further studies are needed to confirm our findings and assess the clinical utility of LH as a non-invasive biomarker for risk stratification and tailed prevention of male infertility.

## Supplementary information

**Additional file 1: Table S1.** Association between serum sex hormone levels and total sperm count^1^. **Table S2.** Association between serum sex hormone levels and sperm concentrations^1^.

## Data Availability

The datasets used and/or analysed during the current study are available from the corresponding author on reasonable request.

## Abbreviations

LH: Luteinizing hormone
FSH: Follicle-stimulating hormone
TT: Total testosterone
E2: Estradiol
SHBG: Sex hormone-binding hormone
BMI: Body mass index
LSM: Least-squares means
CV: Coefficients of variation
