# Supplementary material for: Circulating sex hormone levels in relation to male sperm quality
Source: BMC Urol. 2020 Jul 17;20:101. doi: 10.1186/s12894-020-00674-7 (PMC7367383; doi:10.1186/s12894-020-00674-7)
Supplement: Supplementary file 1 — Additional file 1: Table S1. Association between serum sex hormone levels and total sperm count1. Table S2. Association between serum sex hormone levels and sperm concentrations1. [file 12894_2020_674_MOESM1_ESM.docx]

| **Supplementary Table 1** | | | | |
| --- | --- | --- | --- | --- |
| Association between serum sex hormone levels and total sperm count^1^. | | | | |
| Sex hormone | n | Total sperm count (10^6^) | | |
|  |  | Least-squares mean (95% CI) | *P* | *P* for trend |
| Luteinizing hormone |  |  |  |  |
| Tertile 1 | 112 | 260.9(221.4, 300.4) | Ref | 0.34 |
| Tertile 2 | 114 | 271.4(232.2, 310.5) | 0.71 |  |
| Tertile 3 | 112 | 233.7(194.4, 273.1) | 0.34 |  |
| Follicle-stimulating hormone |  |  |  |  |
| Tertile 1 | 112 | 256.6(217.4, 295.8) | Ref | 0.10 |
| Tertile 2 | 114 | 278.3(238.8, 317.9) | 0.45 |  |
| Tertile 3 | 112 | 231.3(192.0, 270.7) | 0.10 |  |
| Total testosterone |  |  |  |  |
| Tertile 1 | 96 | 257.8(212.1, 303.4) | Ref | 0.91 |
| Tertile 2 | 97 | 252.7(209.2, 296.1) | 0.87 |  |
| Tertile 3 | 97 | 254.0(208.3, 299.7) | 0.91 |  |
| Free testosterone |  |  |  |  |
| Tertile 1 | 96 | 243.6(199.1, 288.1) | Ref | 0.70 |
| Tertile 2 | 97 | 264.5(221.1, 307.9) | 0.51 |  |
| Tertile 3 | 97 | 256.1(211.9, 300.4) | 0.70 |  |
| Total estradiol |  |  |  |  |
| Tertile 1 | 113 | 2756(236.4, 314.7) | Ref | 0.23 |
| Tertile 2 | 115 | 248.6(209.6, 287.5) | 0.34 |  |
| Tertile 3 | 110 | 241.9(202.1, 281.8) | 0.24 |  |
| Free estradiol |  |  |  |  |
| Tertile 1 | 96 | 268.3(224.7, 312.0) | Ref | 0.24 |
| Tertile 2 | 97 | 265.3(221.8, 308.8) | 0.92 |  |
| Tertile 3 | 97 | 230.9(187.1, 274.6) | 0.24 |  |
| Sex hormone-binding hormone |  |  |  |  |
| Tertile 1 | 112 | 275.9(234.0, 317.7) | Ref | 0.21 |
| Tertile 2 | 113 | 253.2(213.7, 292.8) | 0.45 |  |
| Tertile 3 | 113 | 237.4(97.0, 277.7) | 0.21 |  |
| ^1^ General linear models were adjusted for age at sample collection, BMI (continuous), current smoking (yes or no), and current alcohol consumption (yes or no). *P* for trend was calculated by treating hormone categories as ordinal predictors in multivariate linear regression models. | | | | |

| **Supplementary Table 2** | | | | |
| --- | --- | --- | --- | --- |
| Association between serum sex hormone levels and sperm concentrations^1^. | | | | |
| Sex hormone | n | Sperm concentration (10^6^/ml) | | |
|  |  | Least-squares mean (95% CI) | *P* | *P* for trend |
| Luteinizing hormone |  |  |  |  |
| Tertile 1 | 112 | 88.3(75.7, 100.9) | Ref | 0.06 |
| Tertile 2 | 114 | 87.2(74.7, 99.7) | 0.90 |  |
| Tertile 3 | 112 | 71.2(58.6, 83.7) | 0.06 |  |
| Follicle-stimulating hormone |  |  |  |  |
| Tertile 1 | 112 | 92.1(79.4, 104.7) | Ref | 0.05 |
| Tertile 2 | 114 | 80.5(68.0, 93.0) | 0.20 |  |
| Tertile 3 | 112 | 74.1(61.5, 86.7) | 0.05 |  |
| Total testosterone |  |  |  |  |
| Tertile 1 | 96 | 87.1(72.3, 101.9) | Ref | 0.67 |
| Tertile 2 | 97 | 77.6(63.6, 91.7) | 0.36 |  |
| Tertile 3 | 97 | 82.4(67.7, 97.2) | 0.68 |  |
| Free testosterone |  |  |  |  |
| Tertile 1 | 96 | 80.3(65.9, 94.7) | Ref | 0.94 |
| Tertile 2 | 97 | 87.2(73.1, 101.2) | 0.50 |  |
| Tertile 3 | 97 | 79.6(65.3, 93.9) | 0.95 |  |
| Total estradiol |  |  |  |  |
| Tertile 1 | 113 | 82.7(70.2, 95.3) | Ref | 0.77 |
| Tertile 2 | 115 | 83.8(71.3, 96.3) | 0.91 |  |
| Tertile 3 | 110 | 80.1 (67.3, 92.9) | 0.77 |  |
| Free estradiol |  |  |  |  |
| Tertile 1 | 96 | 80.4(66.2,94.5) | Ref | 0.76 |
| Tertile 2 | 97 | 89.6(75.5,103.7) | 0.36 |  |
| Tertile 3 | 97 | 77.1(62.9, 91.3) | 0.75 |  |
| Sex hormone-binding hormone |  |  |  |  |
| Tertile 1 | 112 | 88.9(75.5, 102.3) | Ref | 0.19 |
| Tertile 2 | 113 | 81.8(69.2, 94.5) | 0.46 |  |
| Tertile 3 | 113 | 76.0(63.1, 88.9) | 0.19 |  |
| ^1^General linear models were adjusted for age at blood collection, BMI (continuous), current smoking (yes or no), and current alcohol consumption (yes or no). P for trend was calculated by treating hormone categories as ordinal predictors in multivariate linear regression models. | | | | |
